# Blimp-1 is a prognostic indicator for progression of cervical intraepithelial neoplasia grade 2

**DOI:** 10.1007/s00432-022-03993-4

**Published:** 2022-04-06

**Authors:** Mayumi Saito, Aarthi Rajesh, Carrie Innes, Rachael van der Griend, Peter Fitzgerald, Bryony Simcock, Peter Sykes, Merilyn Hibma

**Affiliations:** 1 Department of Pathology, Dunedin School of Medicine, University of Otago, P O Box 56, Dunedin, New Zealand; 2 Department of Obstetrics and Gynaecology, University of Otago, Christchurch, New Zealand; 3 Canterbury Health Laboratories, Christchurch, New Zealand; 4 Southern Community Laboratories, Dunedin, New Zealand

**Keywords:** Cervical intraepithelial neoplasia, Biomarkers, High-grade squamous intraepithelial lesion, Prognosis, Human papillomavirus

## Abstract

**Background:**

Progression of cervical intraepithelial neoplasia (CIN) to higher grade disease is associated with persistent human papillomavirus (HPV) infection and an absence of immune-mediated regression. However, the immune microenvironment that distinguishes progression from persistent or regressing lesions has not been well defined.

**Methods:**

A total of 69 patients under the age of 25 with high-risk HPV-positive cytology and biopsy-confirmed p16-positive CIN2 were included in the study. Biopsies were stained using 20 antibodies to a range of immune markers. Based on a 2-year follow-up, samples were analysed in “progressor” (CIN3 +) or “persister/regressor” (CIN1, 2 or normal) groups.

**Results:**

Progression was most strongly associated with Blimp-1 positive cell staining in the lesion (*P* = 0.0019) and with low numbers of infiltrating CD4 cells in the dermal region beneath the lesion (*P* = 0.0022). The presence of CD4, CD8 and T bet-positive cells in the dermal region most strongly correlated with CD11c cells in the persister/regressor but not the progressor group.

**Conclusion:**

High numbers of Blimp-1 + cells in CIN2 lesions may predict progression to more severe disease. Measurement of Blimp-1 may have diagnostic utility for the determination of the need to treat women with cervical pre-cancer.

**Highlights:**

CIN2 progression is associated with high numbers of Blimp-1 positive cells in the lesion. Detection of Blimp-1 in the lesion may have utility as a prognostic test to inform the need to treat CIN2.

**Supplementary Information:**

The online version contains supplementary material available at 10.1007/s00432-022-03993-4.

## Introduction

Cervical cancer is the fourth most common cancer in women globally, with an estimated 604,000 new cases and 342,000 deaths worldwide in 2020 (Sung et al. 2021). Almost all cases of cervical cancer are caused by infection with high-risk human papillomavirus (HPV) types, particularly HPV16 and HPV18. Although there is a highly effective prophylactic vaccine against high-risk HPVs, cervical cancer incidence and deaths remain high globally.

Cervical intraepithelial neoplasia (CIN) or squamous intraepithelial lesions (SIL) are pathologically well-defined pre-cancer stages of cervical cancer that are subcategorized into low-grade (CIN1, LSIL) or high-grade (CIN2/3, HSIL) lesions. Regression of these pre-cancer lesions is frequent in low-grade but less frequent in high-grade pre-cancer, and considered to be immunologically based (Guo and Hua 2020). Around 90% of CIN1 (Loopik et al. 2019), 51–64% of CIN2 (Zhang and Lu 2019; Sykes et al. 2021) and 11–40% of HSIL regresses spontaneously (Insinga et al. 2009) within 2 years. Persistent HPV infection with high-risk HPV types is a significant factor for progression of precancerous lesions to cancer (Saslow et al. 2012).

The incidence of infection with high-risk HPV types is high following sexual debut, and decreases with age (Burchell et al. 2006). In young women, the frequency of regression from HSIL (CIN2) is also significantly higher than in older women (Tainio et al. 2018). HSILs are often treated with local excision of the cervix, but treatment is associated with an increased risk for preterm births and mid-trimester loss (Arbyn et al. 2008; Albrechtsen et al. 2008; Kyrgiou et al. 2016). Observational management has been proposed for women under 25 years with CIN2 to reduce the risk of overtreatment (Massad et al. 2013). Observational management regimes would benefit from a test that predicts the likelihood of progression, enabling informed decision making regarding the need for further treatment.

The prognostic value of quantification of immune cells in lesions is well recognized, as demonstrated by the TNM-Immune classification (Immunoscore), where CD8 and CD3 cell infiltration is measured in conjunction with TNM staging to predict survival for colorectal cancer (Pages et al. 2018). The pre-cancerous stages of cervical cancer could be better defined prognostically if a relationship between one or more immune cell markers and lesion progression or regression could be identified. However, there is currently only limited data reporting the relationship between the local immune response and disease outcome (progression versus regression) for cervical precancer (Koshiol et al. 2014).

In this study, we aim to characterise the immune microenvironment in CIN2 and in the dermal region beneath the lesion in high-risk HPV, p16 and Ki67-positive individuals. We will evaluate the immune cell populations and markers in relation to disease outcome (progression to CIN3 + versus persistence/regression during a 24-month follow-up), to identify markers that may be used to predict prognosis in pre-cancerous lesions of the cervix.

## Materials and methods

### Ethics

This study was part of a larger multicentre trial, the PRINCess study, which was designed to evaluate observational management of CIN2 in women < 25 years (Sykes et al. 2016). The PRINCess study clinical trial (ANZCTR number ACTRN12611000547943), was approved on April 14, 2010, by the Multi-region Ethics Committee (Ethics reference: MEC/09/07/079) and had site-specific local authorization.

### Participants

Subjects were participants in the PRINCess study, which ran over the course of 24 months. Inclusion criteria for the subset of women in this study were biopsy-confirmed and p16-positive CIN2 and a high-risk HPV-positive DNA test on a cytological sample at baseline. Women were followed up with repeat cytology and biopsies at 6-monthly intervals and, based on clinical outcome during the 2-year follow-up term, were divided into ‘progressors’ to histologically confirmed CIN3 + (34 cases)’ or ‘persisters/regressors’ (histologically confirmed persistent CIN2 or regression to a lower grade or normal; 35 cases). Confirmed regression was defined as colposcopy, histology (where available), and cytology findings of CIN1 or less on two consecutive colposcopy visits at least 6 months apart. Regression was not confirmed if a low-grade colposcopic lesion had been visualized but not biopsied. Women were observationally managed but with a clinical endpoint of progression to CIN3 + , at which point they were withdrawn from the observational management and treated.

### Tissue samples

The CIN2 tissues stained and analysed in this study were from the baseline biopsy sample. Formalin-fixed paraffin-embedded (FFPE) serial sections were used. The first and last of the serial sections of each series was confirmed by a second Pathologist, in addition to the original pathological assessment, to contain CIN2.

### Immunohistochemical staining

Four-micrometer‐thick sections were incubated overnight at 37 °C. Slides were deparaffinized in xylene and rehydrated in graded ethanol. After rehydration in distilled water, antigen retrieval was performed by microwaving either in 50 mM tris buffer (pH 9.5) containing 5% urea or in 10 mM sodium citrate buffer (pH 6.0) twice for 10 min at 900 W. Slides were cooled at RT for 20 min before being washed in tris-buffered saline, pH 7.4 (TBS). Non-specific antigen binding sites were blocked with 100 μl of 5% skim milk in TBS for 20 min. Sections were incubated with primary antibodies (Table S1) in a humidified chamber at RT for 90 min. After the three washes in TBS, sections were incubated with the corresponding cross-absorbed secondary antibody (Table S1) at RT for 60 min. For the final 30 min of secondary antibody incubation, 40 μl of a 1:50 dilution of DAPI (Invitrogen) was added to stain cell nuclei. Sections were washed as described above, then incubated in the dark with 0.1% Sudan Black B in 70% ethanol for 5 min, to block autofluorescence. Sections were washed in a stream of TBS, immersed in TBS for 10 min, then mounted with 5 µl of SlowFade™ Diamond (Cat. No. S36972; Invitrogen) and stored at 4 °C prior to imaging. When staining with more than one primary antibody for same host species (Table S2; Panels I, IV, VI and VII), sections were incubated overnight at 4 °C with an excess of unconjugated donkey anti-mouse IgG Fab fragment (Table S1; Jackson ImmunoResearch, West Grove, USA) after the first primary and secondary antibody incubations, prior to the application of the second primary antibody. Sections were washed in TBS as above then incubated with second primary antibody, followed by the steps outlined above. A known positive control slide was included in each batch of staining. p16 and Ki67 staining was carried out by Southern Community Laboratories, Dunedin, New Zealand.

### Evaluation of immunohistochemical staining

Images of the entire tissue for each slide were taken with the DP80 camera (OLYMPUS) with the 20 × objective on a BX53 microscope (Olympus). The CIN2 lesion was defined by a pathologist, and the area of lesion was measured using the tool in the Image J. The dermal area within 500 µm of the epithelial basement membrane was separately measured. The positive cells were counted on each tissue using the cell counter plugin in the Image J software (https://imagej.nih.gov/ij/), and the cells per mm^2^ were calculated. IDO-1, PD-L1 and E1^E4 staining was classified as either negative (absent) or positive (including focal staining) in the CIN2 region only. Ki67 was staining was analysed as has been reported previously, with positive staining in the lower one third, lower two thirds or three thirds of the epidermis being described (Reuschenbach et al. 2012).

### Statistical analysis

For each continuous variable samples were separated into a high group (above the median) and a low group (below the median). Progression to CIN3 + over the 24-month follow-up was assessed for the low and the high groups and survival curves were generated with CIN3 + as the endpoint. A univariate analysis was carried out using the Log-rank (Mantel–Cox) test to calculate *P* values and the Mantel–Haenszel test was used to calculate univariate hazard ratios. Chi-square analysis and Fisher’s exact tests were used to test the relationship between categorical measures. The Mann–Whitney U (M–W U) test was used for pairwise comparisons. The Spearman’s rank-order correlation analysis was performed to calculate the correlation coefficient when comparing different markers. Multiple logistic regression and receiver operating characteristic (ROC) curve analyses were carried out to assess the specificity and sensitivity of selected markers for the detection of progression to CIN3. A Bonferroni-corrected *P* value of < 0.05 was considered statistically significant when carrying out multiple comparisons. All statistical analyses were performed with Prism software (Version 9.0, GraphPad Software, Inc).

## Results

### Patient population

The host immune system plays a key role during cervical carcinogenesis (Doorbar 2006). In this study, we have characterized antigen presenting cells, T and B cells and other specific populations of interest in CIN2 and the dermal region below the lesion in a subset of women under 25 who participated in an observational management trial (the PRINCess study). The regression rate (CIN1 or less) for the PRINCess study was 73% at the 12-month follow-up (Innes et al. 2018). For this study, we over-sampled women from the PRINCess study who had progressed to CIN3 + to generate similar group sizes for the persister/regressor and the CIN3 + progressor groups that were studied here.

A total of 69 cases with high-risk HPV-positive test results and histologically confirmed p16 and Ki67-positive CIN2 were recruited to this study. The participants were separated into the persister/regressor group consisted of (35 cases) or the ‘progressor’ group (34 cases). The characteristics of the study population at baseline are shown in Table 1. Patient age, smoking history and HPV-vaccination status was comparable between the persister/regressor and the CIN3 + progressor groups. The CIN2 baseline lesions in the progressor group were significantly larger (*P* = 0.0039; M–W U test) than the lesions that persister/regressor group. However, a univariate analysis of area with progression to CIN3 + as the outcome did not show any significant association (Table 2).

**Table 1:** Baseline characteristics of persister/regressor and progressor groups

		Persister/regressor (*n* = 35)	Progressor (CIN3 +) (*n* = 34)	Statistical significance
Age	Mean (range)	21.50 (17–24)	21.91 (19–24)	*P* = 0.3159 (M–W U*)
Smoker	Yes	11 (31.4%)	13 (38.2%)	
	No	22 (62.8%)	21 (61.8%)	*P* = 0.5367 (Chi-square)**
	Unknown	1 (2.9%)	–	
	ND	1 (2.9%)	–	
Vaccine	Yes	16 (45.7%)	11 (32.4%)	
	No	8 (22.8%)	8 (23.5%)	*P* = 0.4054 (Chi-square)**
	Unknown	10 (28.6%)	15 (44.1%)	
	ND	1 (2.9%)	–	
Lesion area	Mean (range)	0.2717 (0.0093–1.347)	0.5366 (0.0112–2.148)	*P* = 0.0039 (M–W U*)

*Mann–Whitney U; **Comparison of the categorical responses within the 'smoker' group and within the 'vaccine' group

**Table 2:** Univariate (log rank) and multivariate analyses of progressor (CIN3 +) and persister/regressor CIN2 tissues

	CIN2 lesion			Dermal region beneath lesion		
	Univariate analysis		Multivariate analysis	Univariate analysis		Multivariate analysis
	HR^a^ (95% CI)	*P* value	*P* value	HR (95% CI)	*P* value	*P* value
CD4	1.807 (0.8277–3.944)	0.1375	0.4621	3.882 (1.627–9.260)	0.0022**	0.2015
Tbet	2.412 (1.120–5.194)	0.0244*	0.0082**	1.736 (0.7434–4.055)	0.2024	0.1939
GATA3	0.9065 (0.4007–2.051)	0.8137	0.3736	0.9996 (0.4279–2.335)	0.9993	0.7817
IL17	1.133 (0.5254–2.441)	0.7506	0.6456	0.8855 (0.3787–2.071)	0.7791	0.5085
FoxP3	2.041 (0.8587–4.851)	0.1063	0.0894	1.91 (0.8087–4.511)	0.14	0.5573
FoxP3 + CD4-	0.3835 (0.1680–0.8754)	0.0229*	–	1.837 (0.7794–4.329)	0.1645	0.9470
FoxP3 + CD4 +	1.441 (0.6635–3.131)	0.3557	0.4621	1.91 (0.8087–4.511)	0.1645	0.5049
CD8	0.7771 (0.3495–1.728)	0.5361	0.1588	3.56 (1.512–8.380)	0.0036**	0.2901
Granzyme B	1.014 (0.4714–2.182)	0.9711	0.3584	0.933 (0.3998–2.177)	0.8725	0.6806
CD8 + GranzymeB +	–	–	–	0.7575 (0.3245–1.768)	0.5208	0.5753
Langerin +	0.708 (0.3092–1.621)	0.4141	0.6258	0.5106 (0.2139–1.219)	0.1299	–
Langerin + Fascin +	0.5659 (0.2629–1.218)	0.1455	0.4683	1.192 (0.4205–3.380)	0.7408	–
CD11c +	1.032 (0.4550–2.341)	0.9398	0.3283	2.809 (1.198–6.588)	0.0176*	0.2678
CD32	0.9237 (0.4236–2.014)	0.8419	0.8301	0.6802 (0.3103–1.491)	0.3357	0.3943
CD138	1.193 (0.5474–2.601)	0.6569	0.3250	1.189 (0.5452–2.592)	0.6637	0.9226
HMGB1	0.3692 (0.1699–0.8024)	0.0119*	0.0267*	0.9942 (0.4257–2.322)	0.9892	0.1159
Blimp1	0.2911 (0.1336–0.6345)	0.0019**	0.0006***	0.9308 (0.3963–2.186)	0.8693	0.0547
TSLP	ND	ND	ND^b^	1.276 (0.5409–3.011)	0.5776	0.4858
Area	0.4745 (0.2201–1.023)	0.0572	0.2430	ND	ND	ND

*P < 0.05, **P < 0.01, ***P < 0.001

^a^Hazard ratio

^b^*ND* no data

### Lesion characteristics in persister/regressor and progressor (CIN3 +) groups

Expression of the viral E1^E4 protein occurs at the onset of viral genome amplification and accumulates late in the viral lifecycle (Doorbar 2013). We hypothesized that tissues that were actively infected and E1^E4 positive would not show full thickness staining with the proliferation marker Ki67. Tissues from both groups predominantly showed Ki67 staining in the upper two thirds of the lesion. Only 11 tissues in total stained positive for E1^E4, using the pan-E1^E4 antibody (Table 3). All the E1^E4 positive tissues were Ki67 positive in the lower third or two-thirds only, and not the full thickness of the lesion. There was no significant difference in Ki67 (*P* = 0.3927; Chi-square test) or E1^E4 positivity (*P* = 0.3221; Chi-square test) between the persister/regressor and the progressor groups.

**Table 3:** E1^E4 and Ki67 staining in persister/regressor and progressor (CIN3 +) CIN2 lesions

Persister/regressor (*n* = 35)				Progressor (CIN3 +) (*n* = 34)			
Ki67		E4		Ki67		E4	
1/3*	7 (20.6%)	E4 +	2 (33.3%)	1/3	6 (16.7%)	E4 +	2 (33.3%)
		E4−	4 (66.6%)			E4−	4 (66.6%)
		ND**	1			ND	–
2/3	25 (73.5%)	E4 +	5 (20.8%)	2/3	22 (66.6%)	E4 +	2 (9.1%)
		E4−	19 (79.2%)			E4−	20 (90.9)
		ND	1			ND	–
3/3	2 (5.9%)	E4 +	0 (0%)	3/3	6 (16.7)	E4 +	0 (0%)
		E4−	2 (100%)			E4−	6 (100%)
		ND	–			ND	–

*Ki67 staining in 1/3, 2/3 or 3/3 of the epidermis; **No Data

### The immune microenvironment in CIN2 lesions

A detailed analysis of immune-related markers in the CIN2 lesion and in the dermal region spanning the basement membrane up to 500 µm below the lesion was carried out. Examples of all staining are shown in Suppl. Figure 1. Several of the markers, granzyme B, fascin, GATA3, TSLP and CD138, stained some or all the keratinocytes in the CIN2 region. Except for TSLP, all positive cells in the lesion, including the keratinocytes, were counted for each of the markers. In the case of TSLP, the CIN2 lesions were uniformly stained; therefore, the individual cells were not counted.

**Fig. 1:**
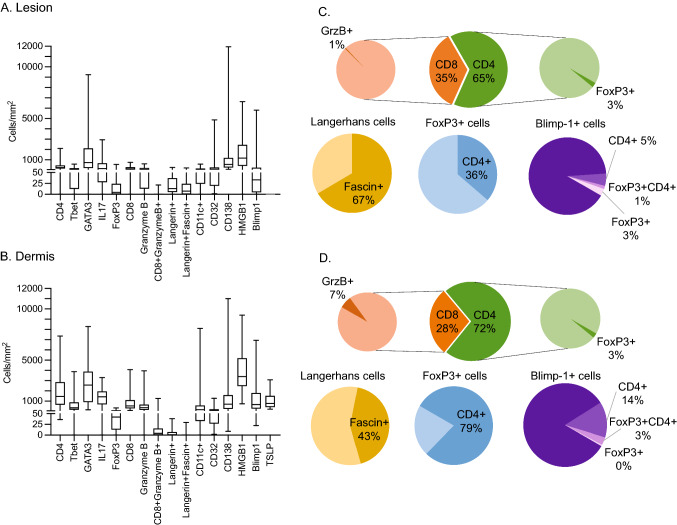
Numbers of cells stained with each of the markers in the lesion and the dermal region beneath the lesion. The positive cells enumerated in the lesion (**A**) and in the dermal region beneath the lesion (**B**). Subsets of cell populations in the lesion (**C**) and the dermal region (**D**) are represented as pie charts

A graphical representation of the cellular infiltrates into the lesion and the dermal region beneath the lesion is shown in Fig. 1. CD4 T cells were present in the CIN2 lesion but were more frequently detected in the dermal region beneath the lesion. Only around 3% of the CD4 cells were also positive for the Treg marker FoxP3, both in the lesion and the dermal region. Parallel staining for T-bet suggested that proportionally around a third of the CD4 cells might be Th1 cells. GATA3-positive staining identifies Th2 cells; however, the number of GATA3-positive cells in the dermal region exceeded the total number of CD4 T cells, due to GATA3 + keratinocytes also being present. In the case of IL-17, the number of cells with positive staining in the nuclei were similar to the total CD4 T cells, suggesting that cells other than CD4 T cells were also being stained. This may be a result of infiltration of other immune cells that express IL-17, including NK T cells, CD8 T cells and neutrophils.

CD8 T cells were detected in CIN2 lesions but were approximately half as frequent as CD4 T cells and were around fivefold less frequent in the lesion than in the dermal region beneath the lesion. Granzyme B expression by the CD8 T cells was measured as an indicator of cytotoxic activity by the cells, however only around 1% of the epidermal CD8 T cells expressed granzyme B, whereas a slightly higher proportion of dermal CD8 cells (7%) expressed granzyme B.

We explored markers that stained antigen-presenting cells (langerin, CD11c, fascin, Clec9A, DC-LAMP) in the CIN2 biopsies. Langerhans cells (LCs), detected using antibodies specific for langerin, were present in the epidermis in low numbers (< 20/mm^2^) and were close to undetectable in the dermis (< 2/mm^2^). Fascin expression in LCs is associated with maturation of LCs and formation of dendritic processes (Ross et al. 1998). The proportion of fascin-positive LCs was higher in the epidermis than in the dermis. We also looked at CD11c, which is a marker for the cDC2 subset in humans. As might be expected, CD11c-positive cells were around four times more frequent in the dermis than in the epidermis, but surprisingly were more commonly found in the epidermis than Langerhans cells. CD11c positive DCs were previously reported to be enriched in anogenital tissues compared to LCs (Bertram et al. 2019). We stained tissues with antibodies for Clec9A to detect cDC1 cells, and DC-LAMP for mature DCs. Both antibodies stained positive control cells in tonsil but no positive cells were detected in the CIN2 biopsies tested in this study.

Antibody secreting cells express CD138, regulating their survival (McCarron et al. 2017). CD138 is also expressed in some tumours and in normal squamous epithelium (Kind et al. 2019), consistent with the full thickness staining of the epidermis that we observed in this study. The staining pattern of CD138 + cells in the dermal region was for surface-stained infiltrating cells, and the numbers of positive cells was similar to the numbers of CD8 T cells that were found in this region and fewer than the CD4 T cells. We also determined the number of CD32B + cells in the epidermis and dermis, which is an inhibitory surface receptor that can down-regulate B cell function, including antibody production. CD32B + cells were detected in similar numbers in the lesion and the dermal region, around tenfold lower than the numbers of CD138 + cells that were detected in the dermal region.

We stained tissues for several positive and negative immune regulatory proteins. HMGB1 is a widely expressed protein with an alarmin function, initiating inflammation (Yang et al. 2020). Pang et al. (2014) found that elevated expression of HMGB1 was associated with a high recurrence of HPV infection in patients with cervical cancer. In this study, we found that HMGB1 was expressed predominantly in the nucleus of most nucleated cells in the dermis, which are primarily fibroblasts. Staining intensity varied from weak to strong, with around 50% of the cells showing strong positive staining. Staining was also observed in the nucleus of epidermal keratinocytes, but the number of positive cells was only about one fifth of the number observed for the equivalent area in the dermal region.

TSLP is a protein expressed T cells and by suprabasal keratinocytes. Its expression induces the release of T cell attracting chemokines, and DC maturation. We observed a small subset of infiltrating cells (< 5% of all TSLP-positive cells) that were strongly TSLP positive in the dermal region beneath the lesion, in addition to the uniform but weaker staining seen in the lesion itself. The numbers of TSLP positive cells were equivalent to around 40% of the total CD4 and CD8 T cells.

The transcriptional repressor Blimp-1 is a regulator of B and T cell development and differentiation (Fu et al. 2017). Loss of Blimp-1 indicates poor prognosis in B-cell-like diffuse large B-cell lymphoma (Xia et al. 2017), whereas Zhu et al. (2017) showed that Blimp-1 inhibited the T cell response in acute myeloid leukemia. Blimp-1-positive cells were identified in the lesion and more frequently in the dermal region beneath the lesion. We were able to characterize Blimp-1-positive cells for FoxP3 and CD4 staining. Around 10% of the Blimp-1-positive cells in the lesion stained for FoxP3 or CD4, and only 1% were triple positive cells. In the dermal region 14% of the Blimp-1-positive cells were CD4 positive, and only 3% of the cells were triple positive (Suppl. Figure 2).

### The relationship between marker expression and progression to CIN3

**Fig. 2:**
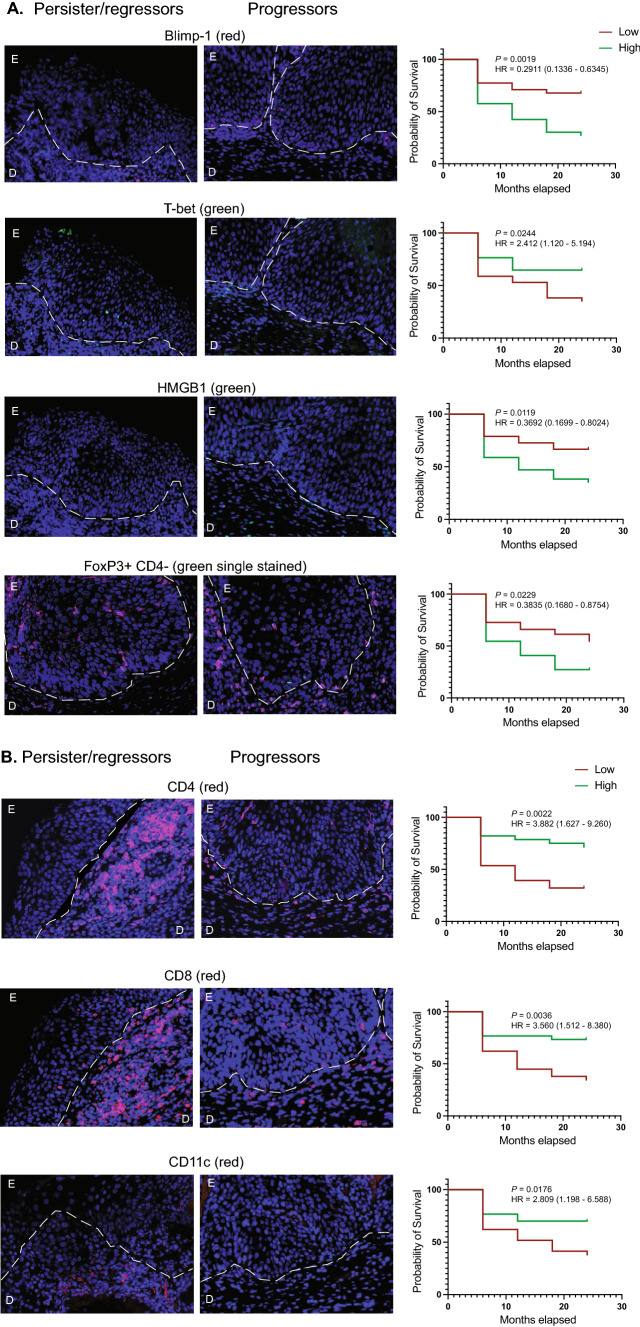
Survival curves for significant markers. Markers that were identified as significantly different in the univariate analysis underwent survival analysis. Counts data were defined as categorical into low (below the population median) or high (above the population median) and survival curves were calculated. Low or high representative images are shown for each survival curve. Images and graphs for lesional staining (**A**) and for staining in the dermal region beneath the lesion (**B**) are shown

The relationship between high or low numbers of the cells identified with each of the markers and progression to CIN3 + over the 24-month follow-up period was assessed (Fig. 2; Table 2). For the univariate analysis, high numbers of cells positive for Blimp-1 (Log-rank test; *P* = 0.002), CD4-FoxP3 + (*P* = 0.02) or HMGB-1 (*P* = 0.01) in the epidermal region (*P* = 0.002) were independently and strongly associated with progression to CIN3. Low numbers of CD4 + (Log-rank test; *P* = 0.002), CD8 + (*P* = 0.004) and CD11c + (*P* = 0.02) cells in the dermal region was also associated with progression to CIN3. Following a Bonferroni correction to adjust for multiple comparisons, high Blimp-1 in the epidermis and low CD4-positive cells in the dermis were confirmed to be significantly associated with progression to CIN3 + .

Expression of immune checkpoint molecules IDO-1 and PD-L1 was also assessed in the CIN2 lesions (Table S3). Overall, less than 20% of the lesions were positive for each of these markers. There were some double positive tissues (5/69), with most double positives (4/35 c.f. 1/34) in the progressor group. There was no relationship between expression of these markers and persistence/regression or progression of disease.

### Correlations between different immune cell subsets in CIN2 that progresses or persists/regresses

**Fig. 3:**
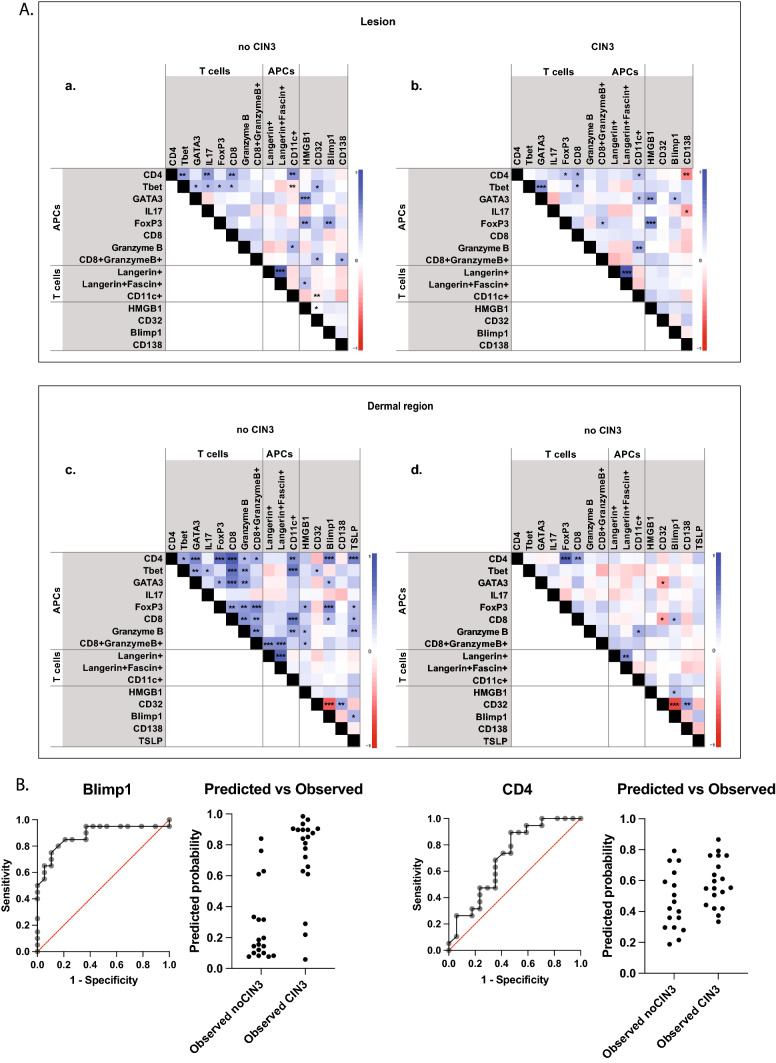
(**A**) The correlation heat map representing *r* values generated from a Spearman’s correlation of the markers with each other for the positive cells in epidermis site for (**A**) no CIN3 and (**B**) CIN3 cases, and in dermis site for (**C**) no CIN3 and (D) CIN3 cases. *P* values are shown on each matrix: *P < 0.05; **P < 001; ***P < 0.001. (**B**) ROC curves and predicted versus observed graphs for lesional Blimp-1 and CD4 + cells in the dermal region beneath the lesion

The relationships between the different cell subsets in the lesion and the dermal region beneath the lesion in the persister/regressor and progressor tissues were evaluated (Fig. 3A). In the epidermis, CD4 cells correlated more strongly with T-bet, IL-17, CD8 and CD11c cells in persister/regressors, compared with the progressor group. Interestingly, T-bet cells inversely correlated with CD11c cells in the persister/regressor group, not in the progressor group. Blimp-1 expression in the lesion did not strongly correlate with other cell populations, except for FoxP3 in the persister/regressor group. In contrast, HMGB1 correlated with FoxP3 positive cells in both groups. The dermal region of the persister/regressor tissues showed the strongest correlations between different cell populations. The related cells were consistent with the key components of an adaptive response. CD4 cells correlated with CD11c-positive cells, CD8 and CD8/granzyme B-positive cells and GATA3, T-bet and FoxP3-positive cells. Blimp-1 and TSLP expression was also positively correlated with CD4 T cells in persister/regressor tissues. This contrasted with the progressor lesions, where CD4 cells correlated only with FoxP3 and CD8 in the dermis.

### Blimp-1 expression in the lesion is significantly associated with progression when adjusted for age, smoking and vaccination status

A multivariate model (multiple logistic regression) was developed to adjust for the effects of age, smoking and vaccination status (Table 2). Blimp-1, HMGB1 and T-bet in the lesion retained statistical significance between the persister/regressor and progressor groups in the multivariate model; however, CD4 T cells in the dermal region were no longer significant following the adjustment, suggesting that one or more of these variables was a confounding factor in the analysis. Following a Bonferroni correction for multiple comparisons, Blimp-1 expression in the lesion retained the significant association with survival when adjusted for confounding factors.

An ROC analysis was carried out as part of the multivariate analysis and the ROC curves showing the sensitivity and specificity of Blimp-1 and CD4 expression in the lesion and the predicted/observed graphs are shown (Fig. 3B). Based on the median cut-off, the separation of samples on high or low expression of Blimp-1 correctly predicted 85% of CIN2 that would progress to CIN3 + and 79% of CIN2 cases that remained CIN2 or regressed to CIN1 or normal, giving Blimp-1 a negative predictive power of 83% and a positive predictive power of 81%.

## Discussion

In this study, we tested the hypothesis that immune markers can predict disease outcome in young women with CIN2. From the analysis of CIN2 lesions obtained from the PRINCess observational trial, we determined that high numbers of Blimp-1-positive cells the CIN2 lesion was associated with subsequent progression to CIN3 in 17 out of the 20 individuals who progressed to CIN3. This is the first report of the transcriptional repressor protein Blimp-1 and its association with progression of CIN2 lesions. This association suggests that Blimp-1 has a role either in advancing the severity of dysplasia in the infected keratinocytes directly, or in the regulation of immune cells activated in response to HPV antigens expressed in the CIN2 lesion. High levels of Blimp-1 mRNA are associated with poor overall survival in some cancers, and improved overall survival in other cancers (Shen et al. 2020). Blimp-1 has been detected in lung and breast cancer cell lines and mRNA expression in a range of cancers has been reported (Yu et al. 2012). Blimp-1 promotes a migratory phenotype in cell lines and is expressed on highly metastatic pancreatic ductal adenocarcinoma (Chiou et al. 2017). Additionally, Blimp-1 regulates peripheral T cells by attenuating proliferation and survival (Boi et al. 2015), it is required to maintain tolerogenic DCs in female mice (Kim et al. 2011), and is expressed in effector regulatory T cells in the mouse (Cretney et al. 2011). Blimp-1 suppresses IL-2 and IFN-γ and is essential for the production of IL-10 by effector regulatory T cells (Fu et al. 2017; Kallies et al. 2006).

We found that Blimp-1-positive cells were strongly correlated with the presence of CD4 T cells and FoxP3-positive cells in the persister/regressor group CIN2 lesions but not in CIN2 lesions that went on to progress to CIN3. However, this is not a consequence of cells double or triple stained with FoxP3, CD4 or both antigens in conjunction with Blimp-1. Considering the multiple immune regulatory functions of Blimp-1 on immune cells, it will be important to establish a more detailed phenotype of the more than 80% of Blimp-1-positive cells that have infiltrated into the CIN2 lesions in this study that are not FoxP3 or CD4 positive to establish the contribution of these cells in CIN3 + progression.

We found that a low CD4 T cell count in the dermal region beneath the CIN2 lesion was associated with progression to CIN3 + ; however, the statistical association was lost when an adjustment for age, smoking and vaccination status was made. The age variability between the groups was limited; however, the CD4 T cell response and infiltration into the lesion could be influenced by vaccination status, and a greater number of women were known to be immunized in the persister/regressor group (46%) than in the progressor group (32%). A recently reported meta-analysis reported five studies showing no association between CIN lesion regression and infiltration of CD4 T cells, and one study that reported low CD4 T cell numbers and recurrence (Litwin et al. 2021). Although we did see an association between low CD4 in the dermal region and progression, this may have been influenced by previous vaccination of some of the participants in the study.

The key findings for this study in relation to T cells and the CIN2 immune microenvironment were that CD4 T cells predominated over CD8 T cells in the dermal region below the lesion, that the majority of FoxP3 positive cells in and beneath the lesion were CD4 negative, that only a small proportion of the CD8 T cells were also granzyme B positive. Additionally, the number of GATA3-positive cells overall exceeded the total number of CD4 T cells in the dermal region, suggesting that other cells were expressing GATA3.

There is some variability in the literature regarding the frequency of CD4 and CD8 T cells in CIN. Woo et al. (2008) carried out a prospective longitudinal analysis of 64 women with low grade CIN over 1 year and also reported a cross-sectional analysis of 125 women, including the 64 women and in some cases more than one slide for each woman (total of 184 slides). They reported that the numbers of CD8 T cells were significantly higher than numbers than CD4 T cells in the intraepithelial compartment, and marginally higher in the stromal compartment, in high-grade CIN (Woo et al. 2008). The CD4 numbers in the lesion and in particular in the dermal region beneath the lesion were higher in our study than in some other reported studies (Litwin et al. 2021); however, there are a number of variables that could contribute to that variation including age and vaccination status.

Woo carried out single staining for CD8 and Granzyme B and reported that the ratio of CD8 + and granzyme B + cells was close to one. We carried out double staining using antibodies to CD8 and granzyme B and show here that the majority of the granzyme B cells in the lesion and the dermis below the lesion are not double positive. Granzyme B is expressed by NK cells in addition to CD8 T cells and can be expressed by keratinocytes extracellularly (Hiebert and Granville 2012). In some pro-inflammatory conditions, granzyme B can be expressed by a range of immune cells including CD4 T cells, Tregs, neutrophils and macrophages, in addition to non-immune cells such as keratinocytes (Boivin et al. 2009). We were able to identify staining consistent with keratinocyte expression of granzyme B on CD8 negative cells in some lesional tissue, showing that expression of granzyme B in CIN2 is not only confined to CD8 T cells.

The LAST Guidelines recommend alignment of terminology for HPV-associated squamous lesions to a two-tiered system of low-grade and high-grade squamous intraepithelial lesions (LSIL and HSIL) (Darragh et al. 2013). They also recommend employing the use of p16 staining if a diagnosis of CIN2 is considered. This study was based on histologically defined CIN2, however the diagnosis was followed up with p16 staining and only p16-positive tissues (HSIL by LAST Guidelines) were included in the study. P16/Ki-67 dual-staining has been suggested as a follow-up test following a positive high-risk HPV result. Co-expression of both proteins does not occur in cells in physiological conditions and co-expression is used as a marker for the presence of high-grade CIN (Petry et al. 2011). All the p16 tissues tested here were also Ki-67 positive, consistent with HSIL. Griffin et al. (2015) proposed that E1^E4 staining could be used in conjunction with MCM to further define lesions (Griffin et al. 2015). They reported that 58% of their CIN2 samples were E1^E4 positive, whereas only 16% of the CIN2 samples studied here were positive and reported that p16 staining usually coincided with an absence of E1^E4 expression. Although more E1^E4 positive staining was detected overall in the persister/regressor group compared with progressor lesions, the numbers of E4 positive samples were too few to draw any useful conclusions about its diagnostic utility.

There are several limitations that should be acknowledged regarding this type of study. Although 20 specific antibodies were tested, the combinations of double-stained antibodies were limited. For example, analysis of CD4 subsets would have been enhanced by double-staining with CD4 and subset-specific markers in combination. Immunohistochemistry has the benefit that spatial relationships between cells can be determined; however, cell dissociation and flow cytometric analysis allows more cells to be quantified with multiple markers. This was not feasible for this study, as biopsy tissue was required for clinical diagnosis. Three markers (epidermal T-bet, HMGB1 and CD4-FoxP3 + cells) were flagged as significant in the univariate and multivariate analyses but were not significant following the Bonferroni correction for multiple comparisons. Seresini et al. (2007) reported that T-bet-positive cells infiltrating the lesion were associated with better clinical outcomes following surgical treatment of HSIL (Seresini et al. 2007), and there are several reports of HMGB1 over-expression in cervical cancer (Hao et al. 2008; Pang et al. 2014). In cervical tissues, high FoxP3 high expression was associated with HSIL (CIN2 and 3) and predicted poor overall survival (Zeng et al. 2013). These markers still may be worthy of testing in larger group sizes to confirm their association, or lack of association, with prognosis.

The immune microenvironment in CIN2 biopsies that persist or go on to regress have a phenotype in the dermal region beneath the lesion that is weighted towards cells of the adaptive immune response (CD4, CD8, CD11c-positive cells). In contrast, CIN2 lesions that progress to CIN3 + are characterized by immune regulatory molecule expression, specifically Blimp-1, FoxP3 and HMGB1, and a loss of Th1 cells in the lesion, suggesting a more tolerogenic tissue environment may exist. Being able to predict the likelihood of progression of HSIL would be highly beneficial in decision-making regarding treatment and reducing over-treatment. High lesional Blimp-1 expression was associated with disease progression and should be tested in larger cohorts to confirm its clinical utility.

## Supplementary Information

Below is the link to the electronic supplementary material.

Supplementary file1 (PDF 30 KB)

Supplementary file2 (PDF 79 KB)

Supplementary file3 (PDF 22 KB)

Supplementary file4 (PDF 6016 KB)

Supplementary file5 (PDF 9951 KB)

## Data Availability

The datasets generated during and/or analysed during the current study are available from the corresponding author on reasonable request.
