# Supplementary material for: Blimp-1 is a prognostic indicator for progression of cervical intraepithelial neoplasia grade 2
Source: J Cancer Res Clin Oncol. 2022 Apr 6;148(8):1991–2002. doi: 10.1007/s00432-022-03993-4 (PMC9294030; doi:10.1007/s00432-022-03993-4)
Supplement: Supplementary file 1 — Supplementary file1 (PDF 30 KB) [file 432_2022_3993_MOESM1_ESM.pdf]

Table S1. List of antibodies

	Ab-1	Source	Catalogue no.	Clonality	Clone	Dilution
**Panel I**	FoxP3	abcam	ab20034	Mouse monoclonal	236AE7	1:100
	Blimp1	NOVUS	NB600-235	Mouse monoclonal	3H2E8	1:100
	CD4	abcam	ab133616	Rabbit monoclonal	EPR6855	1:100
**Panel II-1**	Tbet	Cell Signaling	CTE13232S	Rabbit monoclonal	D6N8B	1:50
	GATA3	Cell Marque	CM390M14	Mouse monoclonal	L50-823	1:50
**Panel II-2**	IL-17	abcam	ab79056	Rabbit polyclonal	-	1:50
**Panel III**	CD8	abcam	ab17147	Mouse monoclonal	144B	1:50
	Granzyme	abcam	ab208586	Rabbit monoclonal	EPR20129-217	1:100
**Panel IV**	Langerin	abcam	ab49730	Mouse monoclonal	12D6	1:100
	Fascin	abcam	ab78487	Mouse monoclonal	55K2	1:800
	TSLP	Merck Millipore	ABT330	Rabbit polyclonal	-	1:200
**Panel V**	Clec9A	R&D Systems	AF6049	Sheep polyclonal	-	1:50
	DC-LAMP	Beckman Coulter	IM3448	Mouse monoclonal	104G4	1:25
	CD11c	abcam	ab52632	Rabbit monoclonal	EP1347Y	1:100
**Panel VI**	HMGB1	abcam	ab79823	Rabbit monoclonal	EPR3507	1:100
	CD138	abcam	ab34164	Mouse monoclonal	B-A38	1:500
	CD32B	abcam	ab45143	Rabbit monoclonal	EP888Y	1:200
**Panel VII**	IDO1	Merck Millipore	MAB5412	Mouse monoclonal	10.1	1:100
	pan-specific E4*	a gift from John Doorbar		Mouse monoclonal	FH1.1	1:300
	PD-L1(R)	Biocare Medical	ACI 3171 A	Rabbit monoclonal	CAL10	1:100
**2˚C Ab**	AF488 anti-Mouse IgG	Thermo Fisher Scientific	A11029	Goat polyclonal	-	1:200
	AF488 anti-Rabbit IgG	Thermo Fisher Scientific	A11070	Goat polyclonal	-	1:200
	AF488 anti-Sheep IgG	Thermo Fisher Scientific	713-545-147	Donkey polyclonal	-	1:200
	AF555 anti-Mouse IgG	abcam	ab150118	Goat polyclonal	-	1:200
	AF647 anti-Rabbit IgG	Thermo Fisher Scientific	A21245	Goat polyclonal	-	1:200
**Fab Fragment**	Donkey Anti-Mouse IgG	Jackson ImmunoResearch	715-007-003	Donkey polyclonal	-	1:25
	Donkey Anti- Rabbit IgG	Jackson ImmunoResearch	711-007-003	Donkey polyclonal	-	1:25

*Reactive against the high-risk HPV types 16, 18, 31, 33, 35, 39, 45, 51, 52, 53, 56, 58, 59, 66, 67, and 70
