# Supplementary material for: Blimp-1 is a prognostic indicator for progression of cervical intraepithelial neoplasia grade 2
Source: J Cancer Res Clin Oncol. 2022 Apr 6;148(8):1991–2002. doi: 10.1007/s00432-022-03993-4 (PMC9294030; doi:10.1007/s00432-022-03993-4)
Supplement: Supplementary file 2 — Supplementary file2 (PDF 79 KB) [file 432_2022_3993_MOESM2_ESM.pdf]

Table S2. List of panel specific details

	PAb-1	Dilution	HIER*	2˚Ab-1	Dilution	Fab	Dilution	PAb-2	Dilution	2˚Ab-2	Dilution
**Panel I**	FoxP3	1:100	Tris-Urea (pH 9.5)	AF488	1:200	Fab	1:25	Blimp1	1:100	AF555	1:200
								CD4	1:100	AF647	1:200
**Panel II-1**	Tbet	1:50	Sodium Citrate (pH 6.0)	AF488	1:200						
	GATA3	1:50		AF555	1:200						
**Panel II-2**	IL-17	1:50	Sodium Citrate (pH 6.0)	AF488	1:200						
**Panel III**	CD8	1:50	Tris-Urea (pH 9.5)	AF555	1:200						
	Granzyme	1:100		AF488	1:200						
**Panel IV**	Langerin	1:100	Tris-Urea (pH 9.5)	AF488	1:200	Fab	1:25	Fascin	1:800	AF555	1:200
								TSLP	1:200	AF647	1:200
**Panel V**	Clec9A	1:50	Tris-Urea (pH 9.5)	AF488	1:200						
	DC-LAMP	1:25		AF555	1:200						
	CD11c	1:100		AF647	1:200						
**Panel VI**	HMGB1	1:100	Tris-Urea (pH 9.5)	AF488	1:200	Fab	1:25	CD138	1:500	AF555	1:200
								CD32B	1:200	AF647	1:200
**Panel VII**	IDO1	1:100	Tris-Urea (pH 9.5)	AF488	1:200	Fab	1:25	pan-specific E4	1:300	AF555	1:200
								PD-L1	1:100	AF647	1:200

*HIER: Heat-Induced Epitope Retrieval
