# Supplementary material for: Blimp-1 is a prognostic indicator for progression of cervical intraepithelial neoplasia grade 2
Source: J Cancer Res Clin Oncol. 2022 Apr 6;148(8):1991–2002. doi: 10.1007/s00432-022-03993-4 (PMC9294030; doi:10.1007/s00432-022-03993-4)
Supplement: Supplementary file 3 — Supplementary file3 (PDF 22 KB) [file 432_2022_3993_MOESM3_ESM.pdf]

Table S3. Expression of PD-L1 and IDO-1 in CIN2 dysplastic cells

		**Persister/Regressor (n=34)**	**Progressor (CIN3+) (n=34)**	Fisher's exact test
**PD-L1**	Positive	6 (17.6 %)	6 (17.6 %)	*P* > 0.9999
	Negative	28 (82.4 %)	28 (82.4 %)	
**IDO-1**	Positive	5 (15.2 %)	11 (32.4 %)	*P* = 0.1517
	Negative	28 (84.8 %)	23 (67.6 %)	
