# Supplementary material for: Blimp-1 is a prognostic indicator for progression of cervical intraepithelial neoplasia grade 2
Source: J Cancer Res Clin Oncol. 2022 Apr 6;148(8):1991–2002. doi: 10.1007/s00432-022-03993-4 (PMC9294030; doi:10.1007/s00432-022-03993-4)
Supplement: Supplementary file 4 — Supplementary file4 (PDF 6016 KB) [file 432_2022_3993_MOESM4_ESM.pdf]

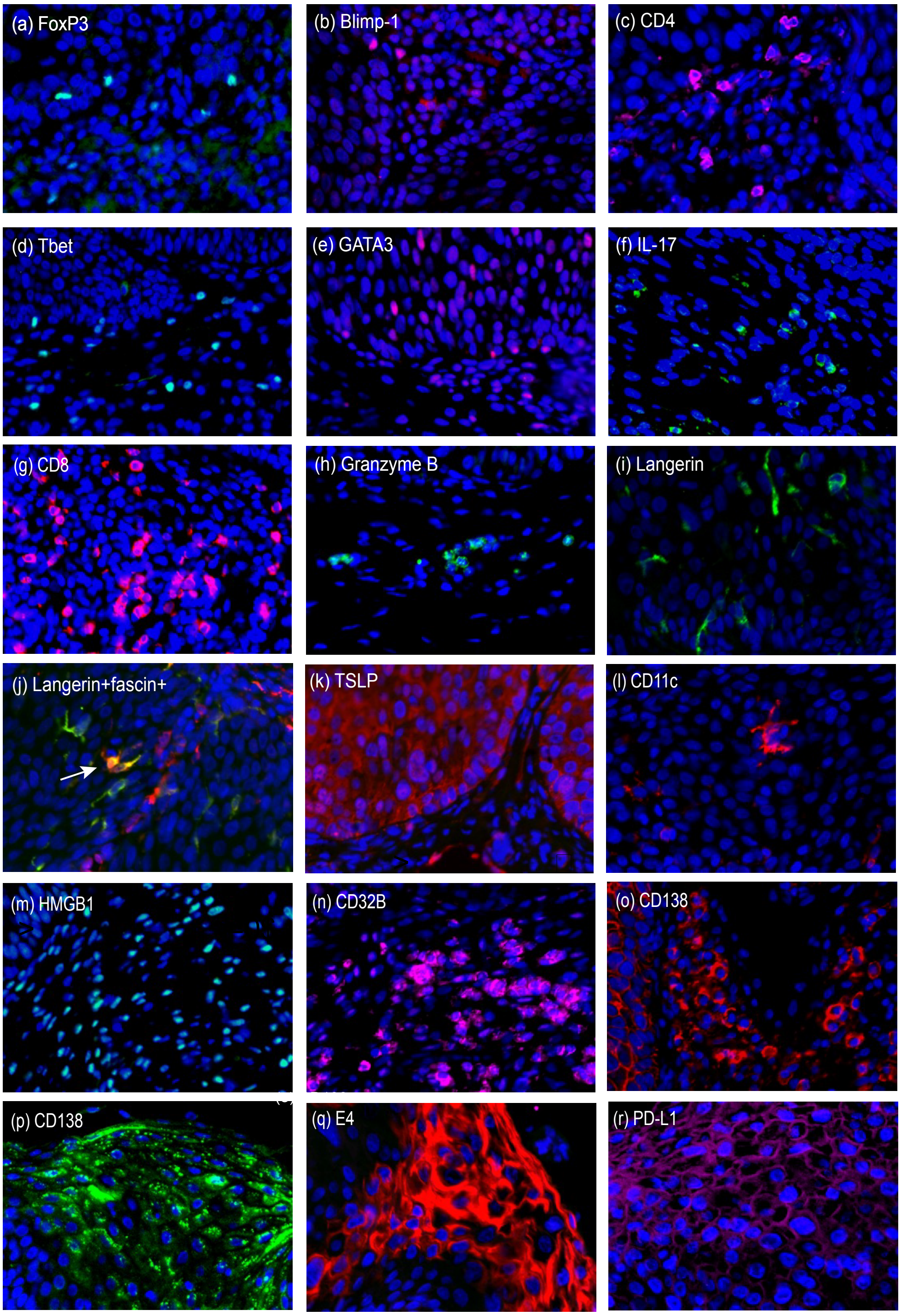

Supplementary Figure 1: Examples of the staining patterns for each of the antibodies on cSCC tissue is shown.(a) FoxP3 (green); (b) Blimp 1 (red); (c) CD4 (red); (d) Tbet (green); (e) GATA3 (red); (f) IL-17 (green); (g) CD8 (red); (h) Granzyme B (green); (i) Langerin (green); (j) Langerin+Fascin (red+green); (k) TSLP (red); (l) CD11c (red); (m) HMGB1 (green); (n) CD32B (red); (o) CD138 (red); (p) CD138 (green); (q) E4 (red); (r) PD-L1 (red).
