# Supplementary material for: Blimp-1 is a prognostic indicator for progression of cervical intraepithelial neoplasia grade 2
Source: J Cancer Res Clin Oncol. 2022 Apr 6;148(8):1991–2002. doi: 10.1007/s00432-022-03993-4 (PMC9294030; doi:10.1007/s00432-022-03993-4)
Supplement: Supplementary file 5 — Supplementary file5 (PDF 9951 KB) [file 432_2022_3993_MOESM5_ESM.pdf]

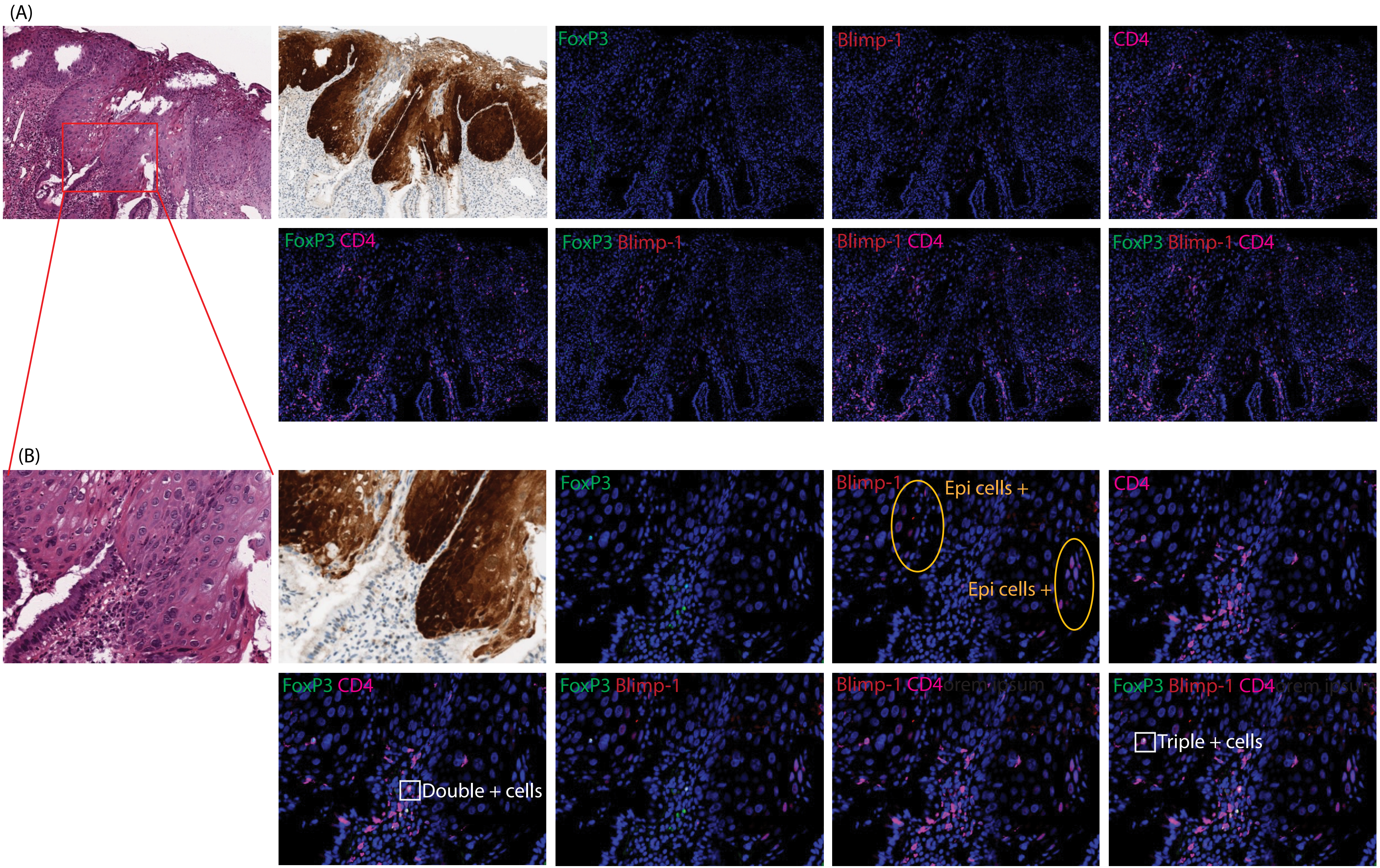

Figure S2: An example image (A) and a close up (B) is shown of the FoxP3, Blimp-1 and CD4 staining panel. Examples of single, double and triple stained cells are shown.
